# Trabectedin Drug Holiday and Rechallenge in Soft Tissue Sarcomas: Report of 4 Cases and Literature Review

**DOI:** 10.3389/fonc.2019.00553

**Published:** 2019-07-09

**Authors:** Francesco Pierantoni, Marco Maruzzo, Antonella Brunello, Benedetta Chiusole, Grazia Pusole, Elisabetta Bezzon, Umberto Basso, Vittorina Zagonel

**Affiliations:** ^1^Medical Oncology 1 Unit, Department of Oncology, Istituto Oncologico Veneto IOV IRCCS, Padova, Italy; ^2^Radiology Unit, Department of Imaging and Medical Physics, Istituto Oncologico Veneto IOV IRCCS, Padova, Italy

**Keywords:** soft-tissue sarcoma, Trabectedin, drug holiday, re-challenge, maintenance therapy

## Abstract

Soft tissue sarcomas are rare neoplasms, with a high mortality rate. Few drugs are available for the treatment of patients affected by metastatic sarcomas, who still have a 5-years survival rate lower than 20%. However, some of the more recent therapies can obtain long lasting responses in a portion of patients, such as Trabectedin. We analyzed four such cases treated at our Institute after progression to an anthracycline based regimen. In each case a therapeutic pause was proposed after at least 6 months of therapy with Trabectedin and in three out of four patients a re-challenge was proposed at progression, achieving again disease control or response. In two cases oligo-progressive sites were treated with localized therapies as stereotactic radiotherapy, delaying the systemic treatment re-start. In this article the reports of the patients involved are presented with a concise review of the relevant literature. Our findings support the favorable safety profile of Trabectedin and the feasibility of drug holidays, which should be at least discussed with the patient.

## Introduction

Trabectedin is an anti-neoplastic drug originally isolated from *Ecteinascidia turbinata*, a sea squirt. The drug exerts its anti-neoplastic activity by binding the minor groove of DNA during replication, causing double strand breaks in the double helix. Moreover, Trabectedin has been found to have a pleiotropic mechanism of action in regulating the inflammatory mediators in the tumor micro-environment (1). This effect is possibly achieved by selective inhibition of the production of pro-inflammatory cytokines and chemokines such as interleukin-6 (IL-6), chemokine ligand 2 (CCL2), matrix-binder protein pentraxin 3 (PTX3), and vascular endothelial growth factor (VEGF) (2). In addition, Trabectedin depletes macrophages in tumor tissue and macrophage targeting appears to be a key component of its anti-neoplastic activity (3).

Trabectedin has been approved by EMA and FDA for the treatment of patients with advanced soft- tissue sarcoma (STS) who have already been treated with anthracycline-based chemotherapy or are ineligible for such regimens (4, 5). Major efficacy has been especially demonstrated for the treatment of leiomyosarcoma and liposarcoma, even if there is evidence of activity also against synovial sarcoma and other subtypes of translocation-related sarcomas (6, 7). Trabectedin is administered at the dose of 1.5 mg/m^2^ every 3 weeks in a continuous infusion across 24 h until unacceptable toxicity or disease progression. Median treatment duration in clinical trials is 3–4 months (4, 5), but some patients may experience prolonged stabilization of disease, and for this particular group the option of a therapeutic pause could be considered.

Trabectedin has been shown to retain activity in patients who were re-treated after progression during a therapeutic pause (8). However, continuous therapy demonstrated improved progression free survival (PFS) vs. discontinuation after six cycles in the T-DIS randomized phase II study, while overall survival (OS) was not significantly different (9). Here we report on four patients who were treated with Trabectedin for a long period of time followed by a treatment pause. In three cases the disease showed to be responsive to a rechallenge with the same drug after progression. All the patients consented to this report.

## Case Report 1

**Figure 1:**
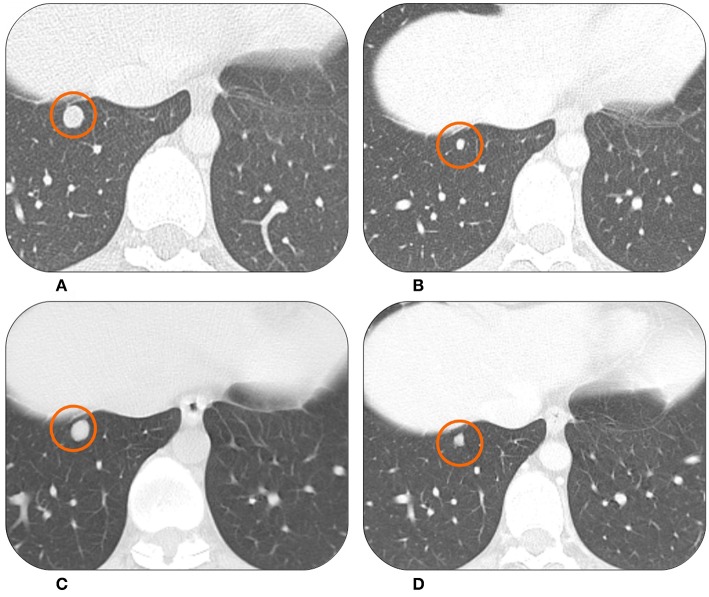
The CT scan of this patient showed lung metastases from synovial sarcoma at the baseline in February 2015 **(A)**. At the first radiological evaluation in April 2015 there was a partial response **(B)**. The patient was on drug holiday from June 2016 to April 2018 when the same lesion started to grow again **(C)**. It then showed a good response to Trabectedin re-challenge in July 2018 **(D)**.

The first patient is a young woman, 20 years old at the time of diagnosis of a synovial sarcoma that originated from the soft tissue of the left hip. The neoplastic mass was surgically removed in May 2012, and the histological examination showed a synovial sarcoma with close surgical margins. Consequently, adjuvant chemotherapy with Doxorubicin and Ifosfamide was administered for a total of six cycles. During follow-up, in February 2015, the CT scan showed almost 15 nodules in the lung ranging from 5 to 10 mm (Figure 1A). In the same month the patient started therapy with Trabectedin at the dose of 1.5 mg/m^2^ every 3 weeks. The first radiological evaluation in April 2015 showed a dimensional reduction of all the lung nodules (Figure 1B). The patient continued the therapy, and in July and October 2015 the CT scan confirmed the partial response of the lung lesions.

In February 2016, after 16 cycles, the patient developed hematological toxicity with thrombocytopenia G2 and neutropenia G2. From the 17th cycle the dose was reduced to 1.2 mg/m^2^ (80% of the expected dose). In June 2016, after 20 cycles, the CT scan still showed stable disease. The patient asked for a therapeutic pause in order to complete her university studies and recover from the treatment's toxicity. Therefore, she started a follow-up program with a CT scan every 3 months.

In September 2017 (13 months after last dose) two lung nodules appeared to be increasing. After a multidisciplinary discussion with dedicated surgeons and radiotherapists it was decided to treat the two lung lesions with stereotactic radiotherapy. The treatment was well-tolerated. However, 6 months later, in March 2018 the radiologic findings showed disease progression with multiple, new lung metastases (Figure 1C). In April 2018, after a new multidisciplinary discussion, it was decided to restart systemic therapy with Trabectedin. The subsequent radiological evaluations in July and October 2018 showed stable disease with signs of response in some nodules (Figure 1D). The patient is continuing Trabectedin, without new significant adverse events. In the meantime, she successfully graduated and started her first work experience.

## Case Report 2

**Figure 2:**
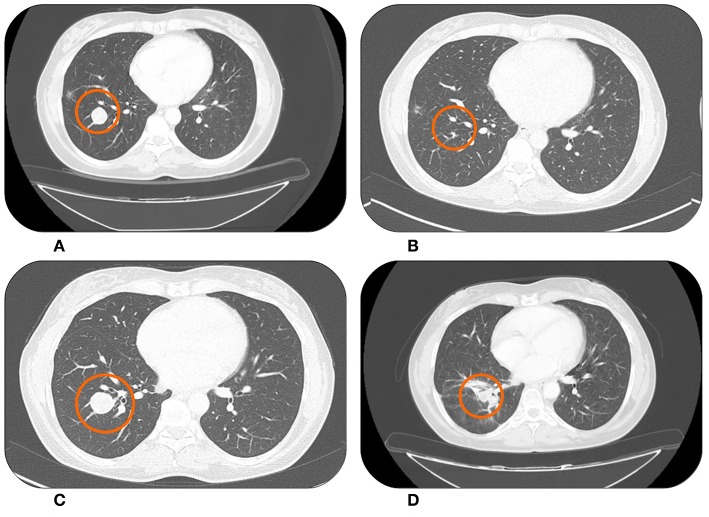
Lung metastases from uterine leiomyosarcoma right before starting Trabectedin in November 2015 **(A)** showing an almost complete response after treatment in February 2016 **(B)**. Systemic therapy was then suspended for a drug holiday. During this period the lesion began to grow again in December 2017 **(C)**. However, in March 2018 after Trabectedin re-challenge a new partial response was observed **(D)**.

The second patient is a woman who was 44 years old at time of the diagnosis of uterine high grade leiomyosarcoma following radical hysterectomy (July 2011). She was referred to our Department in October 2011 when bilateral lung metastases were detected. She underwent a first line chemotherapy with Doxorubicin and Dacarbazine for six cycles, with a very good radiological partial response. In June 2013 the patient received second line therapy with Gemcitabine, due to lung progression. The treatment was stopped in January 2014, after nine cycles, for a drug holiday. In November 2015 a new lung and soft tissue (gluteus muscle) progression occurred (Figure 2A). As third line therapy she received Trabectedin. Overall, the therapy was well-tolerated by the patient. However, she developed hematological toxicity, in particular G3 neutropenia. Consequently, there were some delays of the programmed chemotherapy infusions and the need of granulocyte-colony stimulating factors (G-CSFs). After the introduction of G-CSFs prophylaxis after every cycle the therapy could proceed without delays.

In February 2016 the CT scan documented a partial response (Figure 2B), which was maintained until November 2016 when, according to patient's desire, a drug holiday was started. In July 2017 the CT scan showed a significant dimensional increase of a single lung nodule (Figure 2C). We discussed the case in a dedicated multidisciplinary meeting and decided to treat only the progressive lesion with stereotactic radiotherapy; gaining 6 further months of disease stability. Unfortunately, in December 2017 the patient experienced a multifocal lung progression. Therefore, she re-started Trabectedin with prophylactic G-CSFs and after four cycles, in March 2018, the CT scan documented a partial response (Figure 2D). At the time of this writing the patient is still on therapy maintaining a partial response and without new significant adverse events.

## Case Report 3

**Figure 3:**
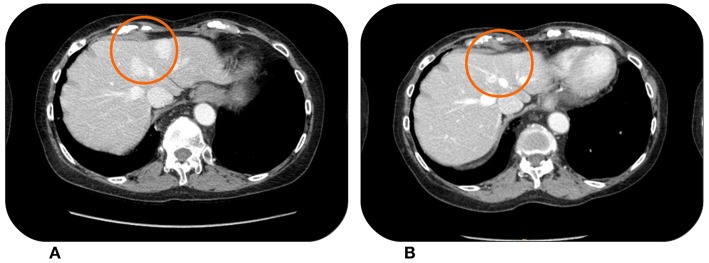
This patient showed hypervascular liver metastases from retroperitoneal leiomyosarcoma at the baseline CT scan in April 2015 **(A)**. The lesions were reduced in both size and vascularisation after treatment with Trabectedin, which was stopped in December 2016 **(B)** for a drug holiday.

The third patient is an elderly woman, aged 78, who underwent the removal of a retroperitoneal mass (July 2014) which was pathologically diagnosed as leiomyosarcoma grade 3. Keeping in consideration the age of the patient no adjuvant treatment was offered. In April 2015 the patient experienced disease progression with multiple lesions in the retroperitoneum, so from May 2015 she received three cycles of pegylated liposomal Doxorubicin. The CT scan in August 2015 showed the appearance of new hepatic lesions (Figure 3A). In the same month she started Trabectedin at the recommended dose of 1.5 mg/m^2^ every 3 weeks. She achieved a partial response in April 2016, with asthenia G2 being the only adverse event. The response was then maintained until December 2016 (Figure 3B), when a therapeutic holiday was proposed to the patient after a total of 22 cycles of Trabectedin. All the next follow-up visits showed stability of disease up to November 2017, when the CT scan documented a stable disease according to RECIST criteria, with only slight increase in some of the lesions. In the following weeks the patient was hospitalized due to complete atrioventricular block, with necessity to implant a pacemaker, so we decided to continue the drug holiday. The hospitalization was prolonged due to episodes of asystolia. At December 2018, the patient was still alive although with an impaired performance status, due especially to her cardiological conditions. Last CT scan performed in November 2018 showed a slow progressive disease, but because of the performance status we decided to not offer active treatment.

## Case Report 4

The last patient is a 43 years old man with myxoid liposarcoma that had his first treatment in April 2003 for what was thought to be a PNET of the popliteal fossa. In fact, he was treated with six cycles of VAC-EI (Vincristine, Actinomycin D, Cisplatin and Ifosfamide, Etoposide) and neoadjuvant radiotherapy (45 Gy) before surgery, achieving a pathological complete response. Seven years later, the patient returned to the Oncology Department for a new supraclavicular mass, which was surgically removed, with a histological diagnosis of low-grade sarcoma. The next year the patient developed two new abdominal masses which were surgically removed. These new surgical specimens and the older histological samples from previous surgeries were then reviewed by an expert pathologist who diagnosed a myxoid round cell liposarcoma in December 2011.

Two years later, in May 2013, the patient experienced a multifocal abdominal progression. The patient was already treated with anthracyclines, so Trabectedin was chosen as therapy for metastatic disease. The therapy was administered at the standard dose of 1.5 mg/m^2^ without any relevant toxicity.

**Figure 4:**
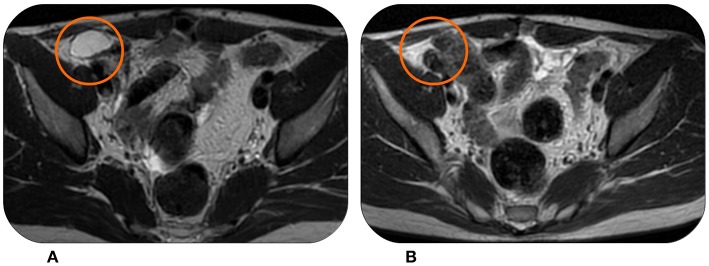
This patient experienced in June 2016 disease progression with multiple peritoneal lesions at MRI **(A)** after being treated with Trabectedin and surgery. However, Trabectedin showed to be effective again, achieving a partial response in February 2018 **(B)**.

The MR scan after nine cycles showed a partial response and, after a multidisciplinary discussion, it was decided to surgically remove the remaining lesions. However, the surgery was not radical. Fortunately, the patient experienced a lasting stability with 25 months of follow-up without relapses. A CT scan showed a new abdominal progression in June 2016 (Figure 4A) with the appearance of multiple peritoneal lesions. We decided for a Trabectedin re-challenge. The patient started at the end of June 2016, and then continued the therapy without significant toxicities. After 18 months of therapy and a new partial response in some of the abdominal nodules (Figure 4B), we proposed a new drug holiday in February 2018. Now, the patient is continuing the second pause and the disease is still stable at follow-up assessments.

## Discussion

In this report we analyzed four patients with advanced sarcoma who were treated for a long period of time (more than 6 months) with Trabectedin, up to the best response, and then started a drug holiday (Table 1). For three of these patients we proposed a Trabectedin rechallenge at progression, achieving signs of response at the subsequent radiological assessments. No unmanageable cumulative toxicities have been detected during the treatment, or at the rechallenge.

**Table 1:** Summary of the clinical outcomes of patients.

**Patient**	**Gender**	**Age at diagnosis**	**Histology**	**Therapies before trabectedin**	**Duration of first trebctedin treatment**	**Reason for drug holiday**	**Drug holiday duration**	**Re-challenge**	**Re-challenge response (RECIST)**
1	F	20	Synovial sarcoma	Doxorubicin + Ifosfamide	16 months	Patient's will	21 months	Y	SD
2	F	44	Leiomyosarcoma	Doxorubicin + Dacarbazine Gemcitabine	13.5 months	Patient's will	13 months	Y	PR
3	F	78	Leiomyosarcoma	Pegylated Liposomal Doxorubicin	16.5 months	Age and comorbidities	24 months	N	N/A
4	M	42	Myxoid round cell liposarcoma	VAC-EI	7 months	Surgery after partial response	30 months	Y	PR

It has been previously reported that a long-term treatment with Trabectedin for advanced STS is safe, feasible, and effective (10). Again, the most recent data from the T-DIS trials are consistent (8).

The role of maintenance chemotherapy in soft tissue sarcomas is still controversial and has been scarcely addressed in a proper manner. Recently, the long-term follow-up results of the randomized phase II T-DIS trial have been reported. The trial confirmed that Trabectedin discontinuation in non-progressive patients with advanced, Doxorubicin-refractory STS after six treatment cycles is associated with rapid disease progression (8). Nevertheless, the PFS outcome from the seventh cycle of treatment resulted in no statistically significant difference between the group of patients who continued treatment and the group of patients who re-started therapy at disease progression. In this sense, it is possible to hypothesize that Trabectedin retains its activity in patients who are rechallenged after disease progression which occurred during a drug holiday. Our experience in the clinical practice setting confirms this activity, showing that Trabectedin is safe and could be effective at progression after a temporary treatment pause. Nevertheless, from a clinical point of view, the proposal of suspending an effective treatment for a drug holiday is controversial and needs to be discussed in detail with our patients. Apart from the results of the T-DIS phase 2 trial, little data are available in metastatic STS. For instance, the role of maintenance of Imatinib in advanced gastrointestinal stromal tumors (GISTs) has been well-established and Imatinib interruption in long-lasting responders results in a high risk of rapid progression (11). On the other hand, the results of a phase III trial with maintenance Ridaforolimus in metastatic STS patients who previously responded to chemotherapy led to a clinically irrelevant improvement in PFS compared with placebo (12). Again, in patients with angiosarcoma the results of a phase II trial reported the lack of benefit of Bevacizumab maintenance therapy (13).

Anyhow, we are learning some lessons from the treatment of other metastatic diseases. Some recent studies in advanced renal cell carcinoma showed that intermittent breaks of Sunitinib or other tyrosine kinase inhibitors (TKIs) treatment could decrease toxicity without compromising the efficacy, and most renal tumors responded after the reinitiation of the same therapy (14–16). Moreover, it has been reported that patients with renal cell carcinoma who reached complete response could safely stop treatment, achieving a significant response rate at rechallenge after disease progression (17). In this setting, the guidelines currently recommend the discussion of the option of a drug holiday instead of treatment continuation for patients with a disease stabilized for at least 12 months. It is clear that STS is a different disease with a wide spectrum of behavior compared to renal cell carcinoma. However, we can at least speculate that a drug holiday is not an uncommon situation in medical oncology and after a long period of treatment, at disease stabilization, it could be an option to be considered in order to improve patients' quality of life. In addition, Trabectedin has several mechanisms of action, and its effect in influencing the composition of the tumor milieu (2, 3) shares some similarities with targeted therapies such as Sunitinib, especially anti-angiogenic activity (18). We can hypothesize that the long-lasting responses to Trabectedin observed may be related to these effects on tumor micro-environment, similarly to what happens with TKIs treatments. Yet, more studies are needed to confirm this assumption.

It is also important to consider that, in slow progressive diseases, the integration with localized treatments such radiotherapy, surgery or interventional radiology procedures can contribute in delaying the rechallenge with systemic therapy, or avoiding shifting to a new line of chemotherapy in many kind of metastatic neoplasms, including STS (19).

Unfortunately, the prognosis of advanced STS is still very poor, and the development of new drugs is struggling. In fact, the enthusiasm for Olaratumab (a novel anti platelet derived growth factor receptor antibody) rapidly deflated after the recent Lilly press release about the results of the phase III trial: Olaratumab in association with Doxorubicin demonstrated no significant increase in overall survival vs. Doxorubicin alone (20). Therefore, it is important for clinicians to use every active drug and procedure at their disposal effectively and efficiently, with the aim of improving the outcomes of patients with this rare disease. Treatment strategies should attempt to find a balance between giving the most active therapies and limiting adverse events in order to improve our patients' quality of life. In conclusion, our findings in long term responder patients support the favorable safety profile of Trabectedin and the feasibility of drug holiday, which should be at least discussed with the patient.

## Data Availability

All datasets analyzed for this study are included in themanuscript and/or the supplementary files.

## Ethics Statement

The patients involved agreed in written form to have their clinical report (including age, gender, medical history, laboratory exams and radiological images) used in an anonymised manner (without name, surname, initials, exact birthdate, ID number) for publication or presentation in congresses.

## Author Contributions

FP, MM, and UB wrote the paper. EB reviewed the radiological images. VZ, AB, and UB supervised and approved the final version of the paper. BC and GP contributed with review of relevant literature, references, and the patients' medical histories.

### Conflict of Interest Statement

The authors declare that the research was conducted in the absence of any commercial or financial relationships that could be construed as a potential conflict of interest.

## References

[1] Larsen AK Galmarini CM D'Incalci M. Unique features of Trabectedin mechanism of action. Cancer Chemother Pharmacol. (2016) 77:663–71. 10.1007/s00280-015-2918-1 26666647

[2] Germano G Frapolli R Simone M Tavecchio M Erba E Pesce S. Antitumor and anti-inflammatory effects of trabectedin on human myxoid liposarcoma cells. Cancer Res. (2010) 70:2235–44. 10.1158/0008-5472.CAN-09-2335 20215499

[3] Germano G Frapolli R Belgiovine C Anselmo A Pesce S Liguori M. Role of macrophage targeting in the antitumor activity of trabectedin. Cancer Cell. (2013) 23:249–62. 10.1016/j.ccr.2013.01.008 23410977

[4] Le Cesne A Blay JY Judson I Van Oosterom A Verweij J Radford J. Phase II study of ET-743 in advanced soft tissue sarcomas: a European Organisation for the Research and Treatment of Cancer (EORTC) soft tissue and bone sarcoma group trial. J Clin Oncol. (2005) 23:576–84. 10.1200/JCO.2005.01.180 15659504

[5] Demetri GD von Mehren M Jones RL Hensley ML Schuetze SM Staddon A. Efficacy and safety of Trabectedin or Dacarbazine for metastatic liposarcoma or leiomyosarcoma after failure of conventional chemotherapy: results of a phase III randomized multicenter clinical trial. J Clin Oncol. (2016) 34:786–93. 10.1200/JCO.2015.62.4734 26371143 PMC5070559

[6] Le Cesne A Cresta S Maki RG Blay JY Verweij J Poveda A. A retrospective analysis of antitumour activity with Trabectedin in translocation-related sarcomas. Eur J Cancer. (2012) 48:3036–44. 10.1016/j.ejca.2012.05.012 22749255

[7] Zanardi E Maruzzo M Montesco MC Roma A Rastrelli M Basso U. Response to Trabectedin in a patient with advanced synovial sarcoma with lung metastases. Anticancer Drugs. (2014) 25:1227–30. 10.1097/CAD.0000000000000158 25075796 PMC4222791

[8] Kotecki N Le Cesne A Tresch-Bruneel E Ray-Coquard I Chevreau C Bertucci F. Impact of Trabectedin interruption and subsequent rechallenge on progression in patients with advanced soft tissue sarcoma: long-term follow-up of the T-DIS trial. Am J Clin Oncol. (2018). 10.1097/COC.0000000000000430. [Epub ahead of print]. 29509592

[9] Le Cesne A Blay JY Domont J Tresch-Bruneel E Chevreau C Bertucci F. Interruption versus continuation of trabectedin in patients with soft-tissue sarcoma (T-DIS): a randomised phase 2 trial. Lancet Oncol. (2015) 16:312–9. 10.1016/S1470-2045(15)70031-8 25680558

[10] Maruzzo M Brunello A Diminutto A Rastrelli M Basso U. Long-term response to first-line trabectedin in an elderly female patient with a metastatic leiomyosarcoma unfit for anthracycline. Anticancer Drugs. (2016) 27:264–7. 10.1097/CAD.0000000000000326 26629769 PMC4736294

[11] Le Cesne A Ray-Coquard I Bui BN Adenis A Rios M Bertucci F. Discontinuation of imatinib in patients with advanced gastrointestinal stromal tumours after 3 years of treatment: an open-label multicentre randomised phase 3 trial. Lancet Oncol. (2010) 11:942–9. 10.1016/S1470-2045(10)70222-9 20864406

[12] Demetri GD Chawla SP Ray-Coquard I Le Cesne A Staddon AP Milhem MM. Results of an international randomized phase III trial of the mammalian target of rapamycin inhibitor ridaforolimus versus placebo to control metastatic sarcomas in patients after benefit from prior chemotherapy. J Clin Oncol. (2013) 31:2485–92. 10.1200/JCO.2012.45.5766 23715582

[13] Ray-Coquard IL Domont J Tresch-Bruneel E Bompas E Cassier PA Mir O. Paclitaxel given once per week with or without Bevacizumab in patients with advanced angiosarcoma: a randomized phase II trial. J Clin Oncol. (2015) 33:2797–802. 10.1200/JCO.2015.60.8505 26215950

[14] Ornstein MC Wood LS Elson P Allman KD Beach J Martin A. A phase II study of intermittent sunitinib in previously untreated patients with metastatic renal cell carcinoma. J Clin Oncol. (2017) 35:1764–9. 10.1200/JCO.2016.71.1184 28113029

[15] Johannsen M Staehler M Ohlmann CH Flörcken A Schmittel A Otto T Outcome of treatment discontinuation in patients with metastatic renal cell carcinoma and no evidence of disease following targeted therapy with or without metastasectomy. Ann Oncol. (2011) 22:657–63. 10.1093/annonc/mdq437 20870911

[16] Mittal K Derosa L Albiges L Wood L Elson P Gilligan T. Drug holiday in metastatic renal-cell carcinoma patients treated with vascular endothelial growth factor receptor inhibitors. Clin Genitourin Cancer. (2018) 16:e663–7. 10.1016/j.clgc.2017.12.014 29428404

[17] Albiges L Oudard S Negrier S Caty A Gravis G Joly F. Complete remission with tyrosine kinase inhibitors in renal cell carcinoma. J Clin Oncol. (2012) 30:482–7. 10.1200/JCO.2011.37.2516 22231040

[18] Dossi R Frapolli R Di Giandomenico S Paracchini L Bozzi F Brich S. Antiangiogenic activity of trabectedin in myxoid liposarcoma: involvement of host TIMP-1 and TIMP-2 and tumor thrombospondin-1. Int J Cancer. (2015) 136:721–9. 10.1002/ijc.29023 24917554

[19] Smolle MA van Praag VM Posch F Bergovec M Leitner L Friesenbichler J. Surgery for metachronous metastasis of soft tissue sarcoma - A magnitude of benefit analysis using propensity score methods. Eur J Surg Oncol. (2019) 45:242–8. 10.1016/j.ejso.2018.06.019 30031674

[20] Lilly Reports Results of Phase 3 Soft Tissue Sarcoma Study of LARTRUVO® Study did not meet the primary endpoints of overall survival (OS) in the full study population or in the leiomyosarcoma (LMS) sub-population; there was no difference in survival between the study arms for either population. There were no new safety signals identified and the safety profile was comparable between treatment arms. Indianapolis, IN: PRNewswire (2019).

